# Favorable physiological and morphological effects of molybdenum nanoparticles on tobacco (*Nicotiana tabacum* L.): root irrigation is superior to foliar spraying

**DOI:** 10.3389/fpls.2023.1220109

**Published:** 2023-08-31

**Authors:** Juanni Chen, Ying Yin, Yunsong Zhu, Kun Song, Wei Ding

**Affiliations:** Laboratory of Natural Product Pesticides, College of Plant Protection, Southwest University, Chongqing, China

**Keywords:** Mo NPs, tobacco, root irrigation, stimulating growth, morphological effects

## Abstract

**Introduction:**

Nano fertilizers can provide efficient solutions to the increasing problem of nutrient deficiency caused by low availability. However, the most important prerequisite is to fully understand whether nanomaterials induce phytotoxicity in plants under a variety of different conditions. The mechanisms underlying interactions between molybdenum nanoparticles (Mo NPs) and plants with respect to their uptake and biological effects on crops are still not fully understood.

**Methods:**

In this study, the impacts of Mo NPs over a range of concentrations (0, 25, and 100 μg/mL) on tobacco (*Nicotiana tabacum* L.) seedling growth were comparatively evaluated under foliar applications and root irrigation.

**Results:**

The results indicated that more significant active biological effects were observed with root irrigation application of Mo NPs than with foliar spraying. The agronomic attributes, water content and sugar content of Mo NPs-exposed seedlings were positively affected, and morphologically, Mo NPs induced root cell lignification and more vascular bundles and vessels in tobacco tissues, especially when applied by means of root irrigation. Moreover, the photosynthetic rate was improved by 131.4% for root exposure to 100 μg/mL Mo NPs, mainly due to the increased chlorophyll content and stomatal conductance. A significant concentration-dependent increase in malonaldehyde (MDA) and defensive enzyme activity for the Mo NPs-treated tobacco seedlings were detected compared to the controls. Significantly improved absorption of Mo by exposed tobacco seedlings was confirmed with inductively coupled plasma mass spectrometry (ICP-MS) in tobacco tissues, regardless of application method. However, the accumulation of Mo in roots increased by 13.94 times, when roots were exposed to 100 mg/L Mo NPs, higher than that under treatment with foliar spray. Additionally, Mo NPs activated the expression of several genes related to photosynthesis and aquaporin processes.

**Discussion:**

The present investigations offer a better understanding of Mo NPs-plant interactions in terrestrial ecosystems and provide a new strategy for the application of Mo NPs as nano fertilizers in crop production.

## 1 Introduction

The most commonly studied engineered nanomaterials (ENMs) have attracted scientists’ attention due to their extremely small size and very high surface area to volume ratio, which allow them to exhibit unique physical and chemical properties, with various reports demonstrating significant potential applications in modern agriculture, such as nano fertilizers, nano pesticides and other nanoforms of agricultural chemical inputs, which can significantly improve resource use efficiency and the quality of agricultural products, contributing to the sustainable development of agriculture (Raliya et al., 2018; Kumar et al., 2023; Periakaruppan et al., 2023). At the same time, biological effects on plants have become a challenging issue and have yet to be explored in detail although high amounts of nanoparticles (NPs) are discharged into ecosystems. The exponential increase in NPs applications across a wide range of industries, most prominently including medical, pharmaceutical, cosmetic, and electronic, resulting in the release of thousands of tons of nanomaterials into the agricultural production system each year (Liu et al., 2022). The global market of NPs expected to exceed $125 billion by 2024 (Wiseguyreports, 2019). Therefore, there is an urgent need to study the potential effects of these nanomaterials on highly exposed biological systems.

Numerous researchers have documented the biological (positive or negative) effects of ENMs on plant growth which depends on plant species tested, the properties of NPs, including size, functional groups, concentrations, as well as application methods of NPs (Gui et al., 2015; Bora et al., 2022). Among these, different application methods have been explored for nano fertilization to achieve the best promotion effect in the literature, including root irrigation, foliar application, stem injection, and seed presowing treatment. For instance, the foliar spray method was the best for Cu NPs to improve stem growth and new leaf appearance of avocado plants compared to other methods, along with complete uptake and translocation of Cu NPs (López-Luna et al., 2023). Kohatsu et al. found that the elemental composition in lettuce roots was mostly affected by the soil irrigation exposure for CuO NPs, while CuO NPs through foliar spray resulted in less disturbance (Kohatsu et al., 2021). For corn (*Zea mays* L.), 500 mg/kg Fe_3_O_4_ NPs applied by soil irrigation for 4 weeks had no impact on plant photosynthesis and biomass (Yan et al., 2020), while another study proposed the potential benefit of the foliar application of 200 mg/L Fe_3_O_4_ NPs to improve the yield and seed quality in cucumbers (*Cucumis sativus* L.) (Gupta et al., 2022). The plant growth-enhancing potential of iron sesquioxide (Fe_2_O_3_) NPs on soybean root growth and photosynthesis has been investigated after foliar spraying, and the increased intensity was far less than that of soil amendments, which may be related to the excessive precipitation of iron ions (Alidoust and Isoda, 2013). Similarly, titanium dioxide (TiO_2_) NPs showed no changes in the germination rate of wheat but exhibited dose dependent effects on wheat when exposed to TiO_2_ NPs in field soil, and different responses of wheat cultivars in loam and sandy loam soils were reported (Zahra et al., 2019; Mustafa et al., 2021).

Mo is an indispensable trace element for plant growth and development and is a major component of Mo flavin protease and 20 kinds of enzymes that catalyze the conversion of nitrates to nitrites (hydrogenase aldehyde oxidase, nitrate reductase). Mo deficiency can lead to the accumulation of nitrate in plants (Moussa et al., 2022). Mo NPs, as nontoxic and inexpensive materials, have great potential in agricultural applications and can be used in the form of nano fertilizers as microelement supplements (Sharma et al., 2021; Huang et al., 2022). To date, a few rudimentary studies have been performed to evaluate the biological effects of Mo NPs on plant growth. Taran et al. found 1μL/g Mo NPs promoted seed germination and activate the antioxidant enzyme activity of chickpea plants which enhanced the adaptability of crops when applied as a presowing treatment (Taran et al., 2016). Meanwhile, 8 mg/L Mo NPs mixed in soil increased the diversity of the rhizosphere microbial community by changing the root secretions of chickpeas (Taran et al., 2014; Thomas et al., 2017). Especially, 100 mg/L biogenic Mo NPs promoted the morphological parameters, nutrients content and ionic balance of wheat plants cultivated in arsenic-contaminated soil (Ahmed et al., 2022). Another study showed that after the introduction of 1mM Mo nanoclusters into the culture medium, the growth of rice roots was promoted but rice and rape root extension was significantly inhibited under higher exposure, maybe accompanied by root necrosis (Aubert et al., 2012; Adhikari et al., 2013). The biometric indices of pea irrigated with 100 ppm Mo NPs were significantly improved and the mineral element content in plants was increased by two times compared to the control (Sutulienė et al., 2021). In hydroponics condition, the increased growth and elevated protein levels in rice seedlings exposed to 100 ppm of α-MoO_3_ and MoS_2_ NPs, indicating hormesis. Meanwhile, tissue-specific distribution of NPs in rice seedlings was observed (Sharma et al., 2021). Nevertheless, these studies have mainly focused on the effects of Mo NPs on the seed germination and root growth of crops under short-term exposure, and more attempts from multiple dimensions need to be made to clarify the biological effects of Mo NPs on plants, including the physiological and molecular responses.

Early seedling stages are the important stages in the plant life cycle, and are preferred for toxicological studies due to their tolerance or sensitivity to external stress (Aslam et al., 1993). Reportedly, vital physiological indicators related to plant health, such as the photosynthetic efficiency, relative water content, and malondialdehyde (MDA) content, and morphological parameters, such as shoot and root lengths and shoot and root weights, can be significantly affected by NPs (Yang et al., 2020b). Photosynthesis is an important energy transfer process in plants and is closely related to stomatal conductance, the photosynthetic rate, the intercellular CO_2_ (Ci) content and the transpiration rate (Tr) (Hoshika et al., 2022). Moreover, metal and metal oxide NPs cause oxidative stress in plants by producing reactive oxygen species (ROS), forcing the plants to evolve an antioxidant defense system as the first line of defense, increasing or decreasing antioxidant enzyme activities (Venzhik and Deryabin, 2023). Although many studies have been performed, plant response varies with the dose applied, plant species, application modes, experimental conditions and the exposure duration of NPs in the growth medium. We can still investigate changes in these important indicators to elucidate the Mo NPs-mediated promotion effect mechanisms, which have been very rarely reported, to our knowledge.

Flue-cured tobacco is an economically important crop in China with a planting area that exceeded 1 million/hm^2^ in 2018, making it an indispensable part of the agricultural ecosystem. In recent years, the application of nanotechnology has made remarkable progress in tobacco disease and pest management, high-quality variety cultivation, soil fertilization, and yield improvement (Frazier et al., 2014). Studies on the biological effects of nanomaterials on the growth and development of tobacco seedlings have not been conducted. This study examines alterations in the various agronomic attributes, biomass, physiology and morphology of tobacco seedlings in a growth matrix under greenhouse conditions, and Mo accumulation in plants and the expression of several genes involved in physiological processes were analyzed in response to foliar applications and root irrigation with Mo NPs. The present study aimed to clarify the ecological behavior of Mo NPs. Most importantly, it is expected to provide a reference for the rational and efficient application of nanomaterials in agricultural production.

## 2 Materials and methods

### 2.1 Chemical materials

Mo NPs (CAS Registry No. 7439-98-7) were purchased from Sigma−Aldrich Chemical Co., Ltd. (St. Louis, USA). The crystalline structure and morphology of the MoNPs were observed using TEM (JEM-2100, JEOL, Japan) and SEM (S-570, Hitachi, Japan), respectively. The particle size was characterized using a Malvern Zetasizer Nano Series (Malvern, United Kingdom).

### 2.2 Plant cultivation and Mo NPs treatment

*Nicotiana tabacum* L. seeds were obtained from the Yunnan Tobacco Research Institute. Before sowing, tobacco seeds were surface sterilized with 75% hydrogen peroxide (H_2_O_2_) for 15 min and sown in a plastic container (9 cm x 8 cm) filled with a standard clay-type matrix. All plastic containers were placed in an artificial climate with a temperature of 27 ± 1°C/18 ± 1°C and a light/dark cycle of 14 h/10 h. The relative humidity was kept at 70-80%. Murashige and Skoog (MS) culture medium without sucrose and agar (Solarbio, Beijing, China), as fertilizer solution, were applied three times at 15 days, 30 days, and 50 days after seeding. The composition of fertilizer solution was shown in **Table S1**. The pH of the nutrient solution was adjusted to 6.8. After the plants grew to the four-leaf stage (approximately 35 d), uniform tobacco seedlings were selected for experimental use.

Mo NPs (Sigma-Aldrich, USA) solutions of different concentrations (0, 25, and 100 μg/mL) were prepared using sterilized distilled water and sonicated in an ultrasonic bath (Bransonic, Danbury) for 1 h to form a homogeneous suspension before experimental use. Two sets of experiments were laid out in a randomized block design. For one group, four-leaf tobacco plants were subjected to foliar sprays of 10 mL of various concentrations of Mo NPs solution. Tobacco seedlings in another group were treated with 10 mL Mo NPs through the root irrigation method. In addition, plants treated with the same volume of sterilized distilled water were used as controls. Tobacco seedlings were treated again 5 days after the first treatment. Twenty plants were randomly selected for each treatment after 25 days, and each treatment was replicated at least three times. All the tobacco seedlings were watered with sterile water at regular intervals during the trial.

### 2.3 Agronomic attributes

After cultivation for 25 days, five exposed tobacco seedlings along with the control were harvested and washed to remove substrates and other residues. The agronomic properties of seedlings were then measured, including seedling height, root length, fresh weight, and biomass. Briefly, following the collection of plants, the fresh weight (FW) of the above-and below ground parts of each plant were immediately determined. Then, seedlings were placed in a hot oven at 105 °C for 30 min and then at 80 °C for another 48 h to achieve constant weight, defined as dry weight (DW) biomass. The seedling moisture content was determined using the following formula:


Moisture content(%)=(FW−DW)/FW×100

### 2.4 Soluble protein and sugar contents in tobacco leaves

The soluble protein content of tobacco seedlings was determined by the Coomassie brilliant blue (CBB) staining method. First, 100 mg CBB was dissolved in 50 mL 95% ethanol, then 100 mL 85% phosphoric acid was added, and the volume was fixed to 1 L. After standing overnight, the solution was filtered with gauze and stored in a brown bottle for later use. Fresh tobacco leaves (0.5 g) subjected to sterilized water and Mo NPs for 25 days were added to a precooled mortar and quickly ground into powder with liquid nitrogen. Then, 5 mL of precooled protein extraction buffer in batches was mixed. The homogenate was transferred to a precooled centrifuge tube and centrifuged at 10,000 rpm at 4 °C for 20 min followed by oscillation for 15 s. Forty microliters of supernatant and 960 μL of water were mixed with 5 mL of CBB G-250 in a precooled centrifuge tube and allowed to stand for 5 min. The absorbance (OD) of the colored supernatant was monitored at 595 nm with an ultraviolet spectrophotometer, and the protein content was determined through the following equation: The amount of protein in the sample (mg/g) = (C× VT)/(vs. × WF × 1000).

The content of soluble sugar in tobacco leaves was determined with anthrone colorimetry as described in previous literature (Koch, 2004).

### 2.5 Determination of the chlorophyll content in tobacco leaves

The chlorophyll content in tobacco leaves was measured according to a previous study (Raliya et al., 2016). After the tobacco plants were exposed to Mo NPs for 25 days under the two application methods, the chlorophyll content in mature leaves of three replicates was measured. Leaves (0.2 g) cut from the same part of tobacco plants were immersed in 10 mL acetone: ethanol extract (V/V = 1:1), and a constant volume of 25 mL was reached after shaking for 14 h in the dark. After the leaves were completely degreed, the supernatant was immediately detected at 645 nm and 663 nm. The formula for calculating the chlorophyll content are as follows:


(1) Ca=12.7* A663−2.69*A645

(2) Cb=22.9* A645−4.68* A663

Chlorophyll(mg/g)=(C×V)/(A×1000)

### 2.6 Photosynthetic rate and stomatal conductance

The photosynthetic rate and stomatal conductance were determined using an LI-6400XT (Licor, USA) in conjunction with an infrared gas exchanger on day 25 after Mo NPs exposure. The machine was equipped with a lamp that was adjusted to a constant quantum flux of 1000 μmoL/m^2^/s and a constant CO_2_ exchange rate of 400 μmoL/moL between two reference sensors. Additionally, a flow rate of 450 μmoL/s was set to control humidity within the chamber.

### 2.7 Measurement of MDA and antioxidant enzyme activity

Fresh leaves (0.5 g) from each treated tobacco seedling were separated, cut and ground in a liquid nitrogen precooling mortar filled with 2 mL of ice-cold potassium phosphate buffer (50 mM, pH 7.8), in an ice bath, to obtain the homogenate. After centrifugation at 10,000 rpm at 4°C for 20 min, the supernatants were collected for experimental use. The level of intracellular lipid peroxidation in the tobacco seedlings was determined based on the content of MDA using assay kits purchased from Nanjing Jiancheng Bioengineering Institute (Nanjing, China). Additionally, the activities of antioxidant enzymes, such as superoxide dismutase (SOD), peroxidase (POD), and catalase (CAT) and defense enzymes including phenylalanine ammonia-lyase (PAL) and polyphenol oxidase (PPO) were measured. Three randomly selected replicates were used.

SOD activity was determined using the nitro-blue tetrazolium (NBT) reduction method, indicated as the absorbance value of superoxide anion production at 560 nm (Berwal and Ram, 2018). POD activity was measured as described by Polle et al. (Polle et al., 1994). POD can act as catalyst to help H_2_O_2_ oxidize guaiacol to red−brown 4-*o*-methoxyphenol, which is an indicator of H_2_O_2_. POD activity was defined as the change rate of the absorbance value of 4-*o*-methoxyphenol at 470 nm. CAT activity and PPO activity were measured according to the method of Taran et al. (Taran et al., 2016).

### 2.8 Leaf, stem, and root cross section observations

For the histopathological study, old leaf (2 cm×2 cm), stem, and root segments (15 mm) were cut out with scissors after treatment with Mo NPs for 25 days and the tissues were immediately fixed in formaldehyde-acetate-ethanol (FAA) for a period of at least 24 h. The sections were rehydrated in two changes of BioDewax and rinsed with 100%, 100%, 75% alcohol and running water for five minutes each. Then, the sections were cut into 5-μm thick sections in a rotary microtome and stained with Safranin O-Fast Green dye (Servicebio, China) for 15-30 s and then subjected to three cylinders of anhydrous ethanol for rapid dehydration. After decolorization by a series of alcohol (50%, 70% and 80%) for 3~8 s, the slides were fixed by green staining for another 6~20 s. The ethanol dehydration process was repeated again, and the sections were placed in xylene for 5 min. Finally, the tissue sections were mounted with neutral balsam and observed under microscope (Nikon Eclipse E100, Japan).

### 2.9 Mo content determination by ICP−MS

After treatment for 25 days, the tobacco seedlings were uprooted carefully and thoroughly washed with deionized water. The leaves, shoots and roots were carefully separated using sterilized scissors and lyophilized with a freeze-dryer. Around 0.2 g of dry tissues were acid-digested after adding 2 mL of pure grade HNO_3_ and 1 mL of 30% H_2_O_2_ in a hot block digestion system (DigiPREP MS, SCP Science) for 50 min at 115°C. The digested solutions were adjusted to 50 mL with DI water and analyzed for total Mo content in the plant tissues by inductively coupled plasma−mass spectrometry (ICP−MS, 163 Agilent 7500ce, Santa Clara, CA).

### 2.10 RT−qPCR analysis of gene expression

After exposure for 25 days, tobacco plant roots exposed to various concentrations of Mo NPs were separately harvested, washed and stored at -80°C until experimental use. Leaf samples (0.1 g) were ground in a frozen mortar using liquid nitrogen and total RNA was isolated using TRIzol (Invitrogen, USA) according to a standard protocol. Afterward, the total RNA was reverse-translated to cDNA using the PrimScript RT Reagent Kit (Takara, Kyoto, Japan).

The RNA was purified using DNA-free DNase Treatment (Promega No. M6101) to eliminate DNA fragments. After the concentration of the cDNA was determined, 2 μL cDNA template was amplified on a Bio-Rad real-time PCR system (Bio-Rad, Hercules, CA, USA) with 40 cycles using a one-step RT−qPCR kit, consisting of 20 μL final reaction volume as described previously, which included 2 μL cDNA template, 7 μL RNase-free water, 7 μL Power SsoFastTM EvaGreen^®^ supermix (Bio-Rad Laboratories, USA), and 1 μL of each forward and reverse primer (Chen et al., 2022). Primer sequences are listed in **Table S2**. The relative RNA expression levels of putative aquaporin (AQP) genes (*NtTIP1, NtPIP1, NtPIP1;1, NtPIP2;1*) and photosynthesis (PS) genes *(rbcL, rbcS, psbA, Lhcb1*) were quantitatively analyzed using CFX manager software, version 1.6 (Bio-Rad, USA). Data analysis was performed using the 2^-△△C T^ method.

### 2.11 Statistical analysis

Statistical analysis of the data was performed with SPSS software version 12.0. The significant differences among the treatment and control groups were determined using the Turkey−Kramer HSD at p = 0.05. All experiments were performed with three replications (triplicates).

## 3 Results

### 3.1 Characterization of Mo NPs

**Figure 1:**
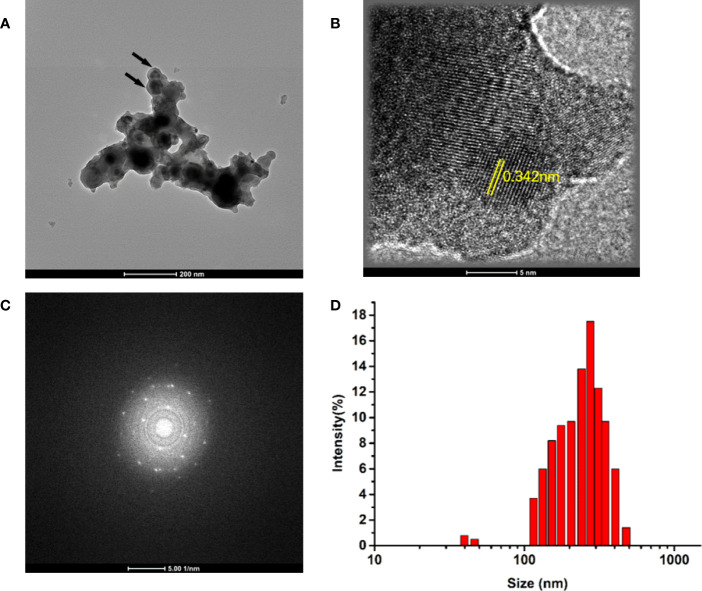
**(A)** Representative transmission electron microscopy (TEM) images of prepared Mo NPs (100 μg/mL); **(B)** Selected area electron diffraction (SAED) patterns; **(C)** High-magnification view of NPs. **(D)** Size distributions of Mo NPs.

The morphology and structure of commercial MoNPs were characterized using transmission electron microscopy (TEM). As shown in **Figure 1A**, TEM demonstrated a spherical-like structure with aggregation and a clearly high crystalline nature as evident from the high-resolution TEM (HRTEM) (**Figure 1B**) and selected area electron diffraction (SAED) images (**Figure 1C**). As presented in **Figure 1D**, the hydrodynamic diameters were analyzed with dynamic light scattering measurements (DLS, Nano S, Malvern) and the mean size of the Mo NPs at a concentration of 100 μg/mL was 331.21 ± 23 nm, which is higher than the size indicated on the product, indicating mass agglomeration of nanoparticles.

### 3.2 Agronomic characteristics of tobacco after root irrigation and foliar spraying of Mo NPs

**Figure 2:**
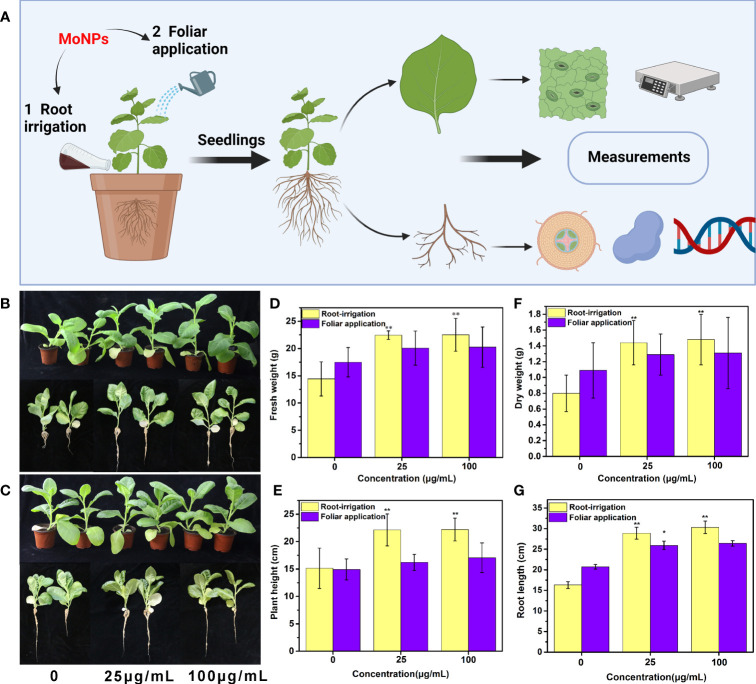
**(A)** Schematic illustration of measurements of tobacco after Mo NPs exposure; Morphological effects of Mo NPs on tobacco seedlings and their roots after root irrigation **(B)** and foliar spraying **(C)** for 25 days; Tobacco agronomic characters of **(D)** fresh weight, **(E)** plant height, **(F)** dry weight and **(G)** root length after different treatment methods. Data are means ± SD (*n* = 5), ∗ and ∗∗ indicate significant differences between the control and Mo NPs treatment at p< 0.05 and p< 0.01 levels according to one-way ANOVA followed by Tukey’s test.

Tobacco plants were exposed to Mo NPs at concentrations ranging from 0-100 μg/mL by root irrigation and foliar spray treatments on the 25th day of plant growth, and then the physiology and morphology of the tobacco seedlings were determined (**Figure 2A**). As shown in **Figures 2B, C**, Mo NPs significantly promoted the growth of the tobacco seedlings after 25 days of root irrigation treatment, while foliar spraying of Mo NPs had no significant impact on the growth of the seedlings. Notably, a concentration-dependent response to the Mo NPs was observed for all the growth indicators of tobacco at the tested exposure doses of nanomaterials under irrigation treatments. Compared with the control group, increases in various growth indicators and biomass were observed for the Mo NPs-treated tobacco seedlings as the concentration of the Mo NPs increased after both foliar and root application. Clearly, for the root irrigation treatment, Mo NPs exhibited a significant promotional effect on tobacco growth, especially on biomass accumulation. The average root length, plant height, fresh weight and dry weight of the tobacco seedlings increased by 31.2%, 46.3%, 55.5%, and 78.8%, respectively, when Mo NPs were applied by irrigation at 25 μg/mL and by 16.5%, 46.6%, 56.0% and 84.0%, respectively, at 100 μg/mL (**Figures 2D–G**). However, for all the agronomic characteristics except root length under 25 μg/mL Mo NPs treatment, no significant difference between the foliar treatment and control was observed, although spraying with Mo NPs promoted the growth of the tobacco seedlings to a certain extent.

### 3.3 Water and chlorophyll content in tobacco upon exposure to Mo NPs

**Figure 3:**
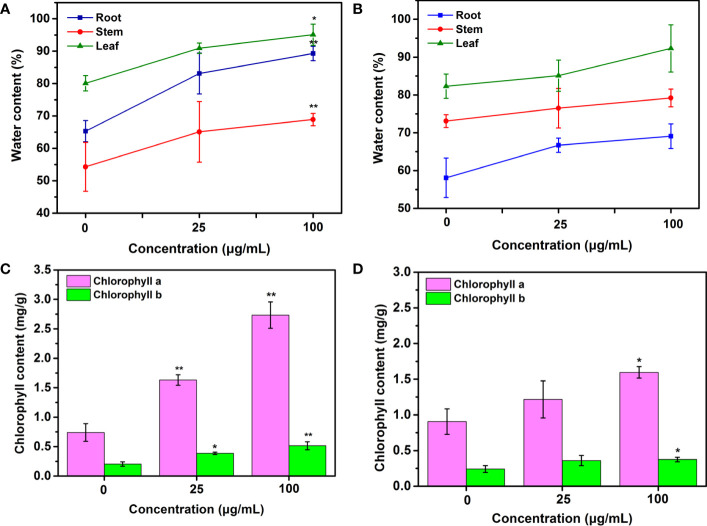
Water, chlorophyll a and chlorophyll b contents of tobacco plants exposed to Mo NPs applied by **(A, C)** root irrigation and **(B, D)** foliar spraying. Representative values are the mean ± SD (*n*=5), ∗ and ∗∗ indicate significant differences between the control and Mo NPs treatments at the p< 0.05 and p< 0.01 levels according to one-way ANOVA followed by Tukey’s test.

Improved water content in tobacco roots, stems and leaves treated with Mo NPs by spraying and irrigation was observed. In the irrigation treatment, the water content in roots, stems and leaves increased by 27.2%, 19.9% and 13.5%, respectively, under 25 μg/mL Mo NPs exposure and by 36.7%,26.9% and 18.7%, respectively, at 100 μg/mL exposure, but no difference was evident between the foliar application and control (**Figure 3**).

Given the close relationship between the chlorophyll content and plant growth, these photosynthetic parameter was measured in this study. As shown in **Figures 3C, D**, the chlorophyll content in tobacco leaves differed depending on the application method. Significant differences between irrigation treatments were observed for both chlorophyll a and b in 25-day-old tobacco leaves in the control and Mo NP-treated groups. The chlorophyll a and b levels increased by 63.12% and 89.02%, respectively, with up to 25 μg/mL Mo NPs and by 269.69% and 152.52%, respectively, after 100 μg/mL Mo NP exposure compared to untreated samples. In comparison, tobacco seedlings exposed to 25 μg/mL foliar-applied Mo NPs displayed a non-significant increase of 34.28% and 49.07% in chlorophyll a and b contents, but these contents increased significantly by 49.07% and 55.24%, respectively, at 100 μg/mL exposure dosages compared with the controls.

### 3.4 Stomatal conductance and net photosynthetic rate

**Figure 4:**
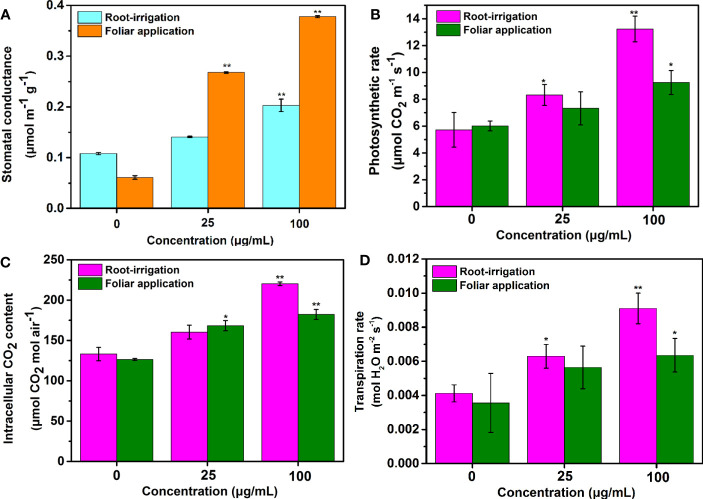
**(A)** Stomatal conductance, **(B)** photosynthetic rate, **(C)** Ci content and **(D)** Tr of tobacco leaves on day 25 after the seedlings were treated with the tested concentrations of Mo NPs (0, 25 or 100 mg/L) under different application methods. ∗ and ∗∗ indicate significant differences between the control and Mo NPs treatments at the p< 0.05 and p< 0.01 levels according to one-way ANOVA followed by Tukey’s test.

As shown in **Figures 4A, B**, the stomatal conductance and photosynthetic rate of treated tobacco gradually increased as the treatment concentration increased, regardless of the application method. However, for plants exposed to Mo NPs under root irrigation conditions, significant correlations between the photosynthetic rate and NPs exposure concentration were observed during the growing period compared to controls. Comparatively, the tobacco seedlings exposed to Mo NPs at 25 and 100 mg/L Mo NPs by irrigation had 45.4% and 131.4% higher photosynthetic rates on day 25 compared with the values (21.7% and 53.4%) obtained in response to spraying. Similarly, when compared to the control, there were significant increases in the Tr (52.3% and 121.4%) and Ci content (20.3% and 65.3%) in plants exposed to 25 and 100 mg/L Mo NPs by irrigation (**Figures 4C, D**). Although, foliar spraying with a low dosage of Mo NPs induced an increase in both photosynthetic parameters, it was not as significant compared with the control, in contrast to the high increase rates of 78.4% and 44.1% in the Tr and Ci contents as the spraying concentration was increased to 100 mg/L Mo NPs. These results indicate that foliar exposure to Mo NPs had slightly weaker effects on the photosynthetic rate and stomatal conductance than soil treatment, which could be due to the lower uptake of NPs.

### 3.5 Lipid peroxidation, and antioxidant enzyme activity in tobacco

**Figure 5:**
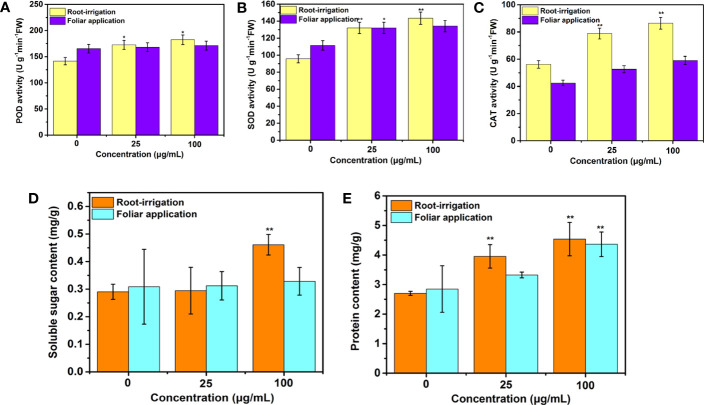
**(A)** POD, **(B)** SOD, and **(C)** CAT activities and **(D)** soluble protein and **(E)** sugar contents in tobacco exposed to 0, 25, and 100 μg/mL Mo NPs for 25 days. Values represent the mean ± SD (*n* = 5). ∗ and ∗∗ indicate significant differences between the control and Mo NPs treatments at the p< 0.05 and p< 0.01 levels according to one-way ANOVA followed by Tukey’s test.

In this study, as shown in **Figure S2**, compared to the unexposed plants, 25-day treatments of Mo NPs at all tested concentrations did not significantly affect the MDA content, indicating that no lipid peroxidation occurred in tobacco leaves during Mo NPs exposure. Furthermore, to investigate how antioxidant systems in tobacco plants respond to Mo NPs exposure, we measured the activity of three important defense enzymes (SOD, POD, and CAT) in different exposure pathways in plant leaves. As shown in **Figures 5A–C**, the relative activities of POD, SOD, and CAT in plants treated with 25 μg/mL Mo NPs were 1.22-, 1.38- and 1.40-fold greater, respectively, than those in the control, respectively, and significantly elevated activity was observed (1.29-, 1.50-, and 1.54-fold) for higher exposure concentrations (100 μg/mL). In particular, in the irrigation treatment with Mo NPs, the activities of the three antioxidant enzymes were notably improved with respect to those in the control, especially CAT activity (**Figure 5C**). However, under foliar spraying conditions, there were no significant differences in defense enzyme activity between the water and Mo NPs treatments, except for SOD and CAT activities under 100 μg/mL Mo NPs exposure (**Figures 5B, C**).

### 3.6 Soluble protein and sugar contents in tobacco

Regarding the sugar level in tobacco leaves, it is noteworthy that 25 mg/L Mo NPs did not exert any significant effect by soil supplementation, whereas the soluble sugar level increased by 58.69% in response to 100 mg/L soil treated with Mo NPs compared with the control. No effects of Mo NPs on the sugar content were observed for foliar application at both concentrations relative to the control (**Figure 5D**). Exposure to Mo NPs by irrigation resulted in a significant increase in the soluble protein content in the tobacco seedlings, reaching 3.95 mg/g and 4.54 mg/g, i.e., improvements of 46.3% and 68.15%, respectively, compared to the control. In contrast, foliar treatment with Mo NPs improved the concentrations of soluble protein only at higher exposure doses (**Figure 5E**). The promotion of the photosynthetic rate can explain these phenomena (**Figure 4**).

### 3.7 Mo NPs uptake and accumulation

**Figure 6:**
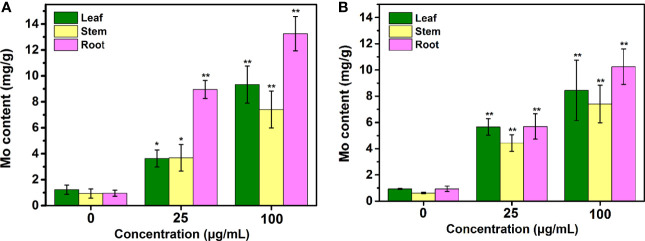
Mo content in various tissues (leaf, stem, and root) of tobacco plants after treatment with Mo NPs by root irrigation **(A)** and foliar spraying **(B)**. Representative values are the mean ± SD (*n* = 5). ∗ and ∗∗ indicate significant differences between the control and Mo NPs treatments at the p< 0.05 and p< 0.01 levels according to one-way ANOVA followed by Tukey’s test.

The Mo contents were examined in the tobacco plants exposed to Mo NPs on day 25. As shown in **Figure 6**, gradual increases in the Mo concentrations in the tobacco plant tissues were observed as the NPs concentration increased, especially in roots, regardless of the application method. The Mo content significantly improved by 6.07-, 7.14-, and 6.06-fold and by 9.08-, 11.95-, and 10.9-fold relative to the control value in leaves, stems and roots, respectively, upon 25 and 100 mg/L Mo NPs foliar exposure, respectively. In contrast to spraying, similar increases in Mo in the tobacco leaves and stems occurred in response to root irrigation with Mo NPs, while the Mo content in the roots changed significantly, almost 9.42 and 13.94 times higher under 25 and 100 mg/L Mo NPs soil exposure, respectively.

### 3.8 Mo NPs induced more vascular bundles and vessels in tobacco tissues

**Figure 7:**
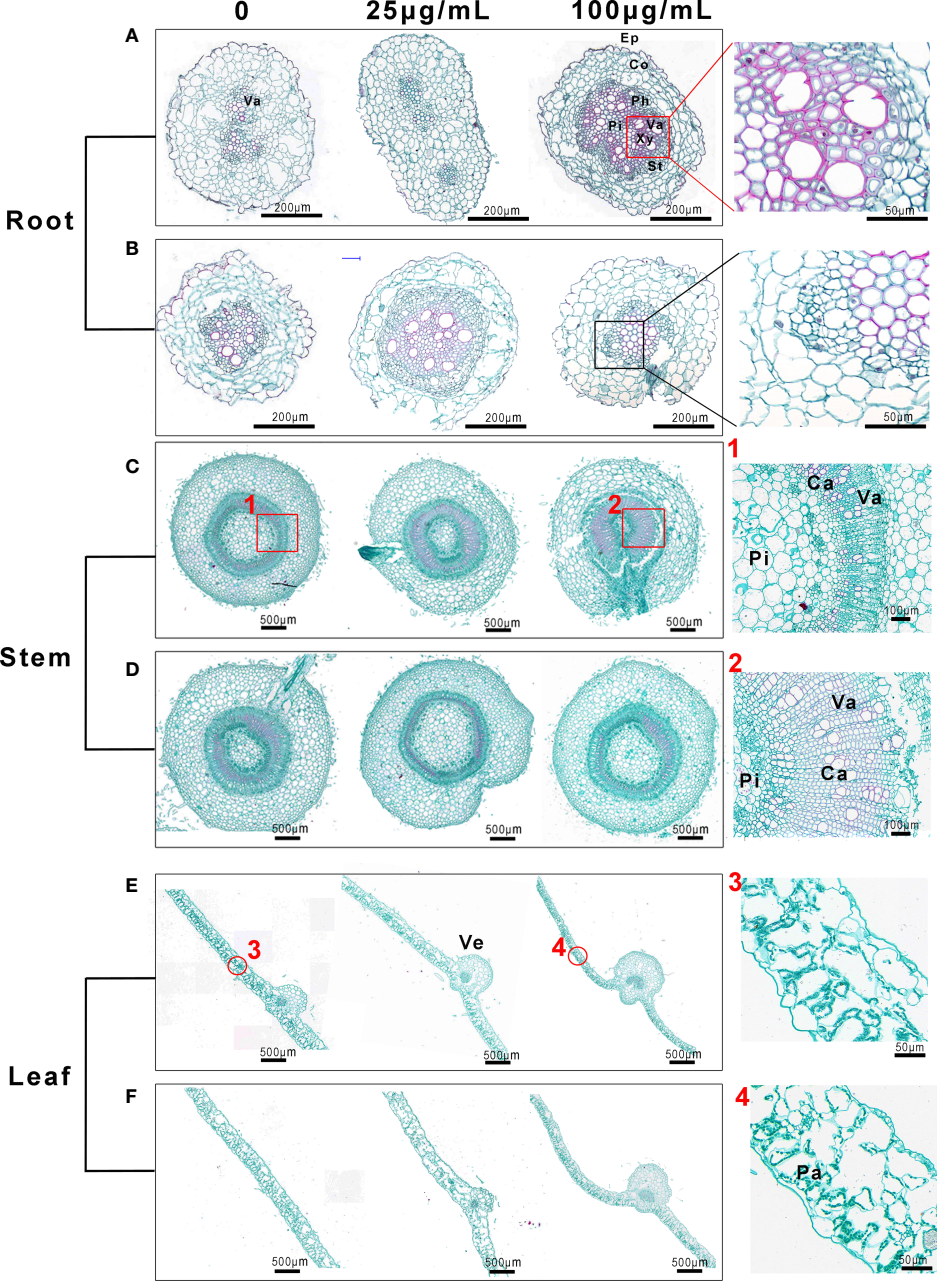
Morphological structures of tobacco roots, stems and leaves, observed with an optical microscope, after root irrigation **(A, C, E)** and spraying **(B, D, F)** with to Mo NPs. The pictures labeled 1 and 2 on the right show the partial magnification of stems in **(C)** and the pictures labeled 3 and 4 show the partial magnification of roots in **(D)**. Cross-sections of tissues from the same position of seedlings treated for 25 days were used for this assay. Ca, catheter; Co, cortex; Ep, epidermis; Fi, fibers; Pa, palisade tissue; Ph, phloem; Pi, pith; St, sieve tube; Va, vascular bundle; Ve, vein; Xy, xylem;.

We further investigated the morphological structures exposed to Mo NPs *via* saffron solid green staining, observed using an optical microscope. As shown in **Figure 7A**, under irrigated conditions a greater area of root tissue was stained red (an indicator of lignification), especially in interfascicular regions, in comparison to the water treatment. The xylem cell walls (red arrows) were obviously strengthened, whereas the root structure of the tobacco seedlings under the foliar application of Mo NPs seemed to be unchanged (**Figure 7B**). There were significant differences in the stem microstructure between the control samples and treatments (**Figure 7C**). Compared with the number of catheters and vascular bundles in the control group, the total cross-sectional area of vascular bundles in the irrigation Mo NPs-exposed stem was larger, and larger catheters and vascular bundles appeared compared with untreated seedlings (Pictures marked 1 and 2). However, these changes did not seem to be observed in tobacco seedlings sprayed with Mo NPs (**Figure 7D**). In the case of leaves, regardless of the route of exposure, the main veins of Mo-treated leaves became thicker compared to control samples, and the cross-sectional areas of vascular bundles, phloem and xylem were also thickened (**Figures 7E, F**). However, there seemed to be no changes in the palisade tissues with the irrigation application of Mo NPs (Pictures marked 3 and 4). Here, the palisade tissues were more regular and tightly arranged after the foliar spraying of Mo NPs (**Figure S3**).

### 3.9 MoNPs upregulated PS and AQP-related gene expression

**Figure 8:**
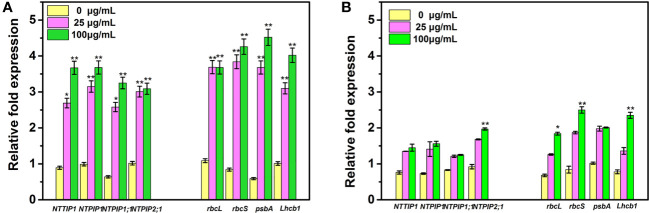
Quantitative real-time transcriptional levels of PS- and AQP-related genes in tobacco seedlings irrigated with Mo NPs **(A)** and sprayed with Mo NPs **(B)** for 25 days. Error bars represent SD (*n* = 3). ∗ and ∗∗ indicate significant differences between the control and Mo NPs treatments at the p< 0.05 and p< 0.01 levels according to one-way ANOVA followed by Tukey’s test.

In the present study, we further analyzed the expression levels of four PS- and AQP-related genes in the tobacco seedlings after the different treatments. As observed in **Figure 8**, under root irrigation treatment for 25 days, all the PS- and AQP-related genes in tobacco under Mo NPs exposure were upregulated in a dose-dependent manner. The expression levels of *NtTIP1*, *NtPIP1*, *NtPIP1;1*, and *NtPIP2;1* in tobacco seedlings exposed to 25 and 100 mg/L MoNPs significantly increased by 3.02-, 3.18-, 4.03-, and 2.95-fold and 4.12-, 3.71-, 5.07-, and 3.02-fold, respectively, compared with the control groups, and *rbcL*, *rbcS*, *psbA* and *Lhcb1* increased by 3.38-, 4.57-, 6.23-, and 3.06- fold and 3.37-, 5.07-, 7.66-, and 3.98-fold under 25 and 100 mg/L Mo NPs. However, after the foliar application of Mo NPs, we found that the expression of only four genes (*NtPIP2;1, rbcL*, *rbcS*, and *Lhcb1*) was greatly upregulated under high concentration exposure. There were no significant differences in the expression of other PS- and AQP-related genes in comparison to control samples.

## 4 Discussion

Notably, in this study, Mo NPs more significantly promoted tobacco seedling growth with root-irrigation application than with the foliar spraying method after 25 d. These results are in accordance with previous reports where strong positive effects on rice and chickpea were observed after Mo NPs treatment. The chickpea seed germination rate was significantly promoted after presowing treatment with Mo NPs, even at the low dosage of 5 mg/L (Adhikari et al., 2013; Taran et al., 2014; Taran et al., 2016). Similarly, the foliar application of Mo sulfide (MoS_2_) NPs to rice obviously promoted rice growth through the enhancement of photosynthesis and the acceleration of cell division and expansion (Li et al., 2018). It is speculated that Mo NPs strengthen the water adsorption capability of soil amendments due to the role of Mo, which contributes to enhancing the water utilization efficiency and osmotic-adjustment ability, probably facilitating the translocation efficiency of a variety of nutrient elements. This finding is consistent with the results of Wu et al.’s study that Mo effectively improved plant water retention and drought resistance (Wu et al., 2014).

Bulk Mo fertilizer, commonly derived from ammonium molybdate and sodium molybdate, is considered an effective strategy to improve crop physiological traits. When wheat is chronically exposed to ammonium molybdate (0.41 kg ha^−1^) in the field, the biomass, grain yield and uptake of P from soil are significantly increased (Rana et al., 2020). In a short-term treatment, seed priming with Mo (0.5 g/L of sodium molybdate) for 8 h increased chickpea yield in a pot study and in field study by 27% and 20%, respectively (Farooq et al., 2012). Other findings indicated that sodium molybdate positively affected leguminous plant (alfalfa and hairy vetch) growth, whether foliar application or soil addition, especially in low-pH soil (5.2), resulting in a significant increase in nodule counts and size, thus accelerating the biological N fixation process (Adhikari and Missaoui, 2017; Alam et al., 2015). However, higher Mo doses (1.0 mg kg^−1^) led to the deterioration of nodule structure (Alam et al., 2015). In contrast, in this study, the concentration of Mo NPs used was far greater than that in the above studies without any phytotoxicity, indicating better biocompatibility. In addition, 50–70% of traditional fertilizers can be lost by means of evaporation, drift, hydrolysis, leaching, runoff, and microbial or photolytic degradation of nutrients, causing the very low use efficiency (Marchiol, 2019). It is worth highlighting that, as nano fertilizer, the physicochemical peculiarities of NPs contribute to the improvement of nutrient delivery efficiency in unique ways, such as specific release, controlled release, moisture release and rapid diffusion (Mikkelsen, 2018).

Some nanomaterials can change the ability to absorb water and regulate the absorption of metal elements in plants due to their hydrophilic properties (Hu et al., 2017). He et al. reported that GO can act as a “water transporter” in the soil, accelerating water absorption, and ultimately promoting the germination of spinach and chive seeds, but GO sheets are not absorbed by the seeds (He et al., 2018). This is slightly different from the mechanism by which MWCNTs puncture the seed coat and promote vegetable seed germination and plant growth by accelerating water uptake by means of enhanced aquaporin gene expression, whereafter they are delivered and accumulate in the plant (Villagarcia et al., 2012; Lahiani et al., 2013).

Chlorophyll, a photoreceptor, channels the energy of sunlight into chemical energy, converting it through the process of photosynthesis. Mo NPs increased the chlorophyll content of tobacco, especially under root irrigation, which is in agreement with the findings of Taran et al., who reported stimulating effects of Mo NPs on the chlorophyll content under soil treatment (Taran et al., 2014), while Yang et al. showed that exposure to all tested concentrations less than 100 mg/kg of Mo nanomaterials had no significant effect on the chlorophyll content in soybean leaves and therefore did not affect the net photosynthetic rate (Yang et al., 2020a). The growth promotion effect of Mo NPs on tobacco after root irrigation treatment is better than that of spraying treatment, which may be due to the better NPs absorption by plant roots. A higher chlorophyll content can improve light absorption and conversion efficiency, thus Mo NPs promote the photosynthesis process of tobacco plants (Hideaki, 2006). Our speculation was verified in this research. It is interesting to note the multiple effects of NPs on the chlorophyll content in a wide range of species (peanut, cotton, corn) in previous studies, including positive and negative effects, but they rarely involved Mo NPs (Adhikari et al., 2013; Thomas et al., 2017; Li et al., 2018; Chen et al., 2022). We speculated that the improved chlorophyll content of tobacco seedlings under Mo NPs exposure may result from the absorption of Mo, which is usually present in the active center of several plant enzymes that catalyze the key steps of nitrogen, carbon, and sulfur metabolism, participate in the chloroplast structure and regulate the production and balance of H_2_O_2_ in plants, promoting the synthesis of chlorophyll in leaves at the seedling stage. The activated mechanisms can be similar to those of MgO NPs (Cai et al., 2018). MgO NPs can be absorbed by plants, and participate in the synthesis and metabolism of magnesium porphyrin compounds in chlorophyll, thereby accelerating the photosynthesis of watermelon and tobacco plants, and even enhancing the resistance of plants to soilborne diseases (Wang et al., 2013).

Photosynthesis is an essential process by which green plants and certain other organisms transform light energy into chemical energy stored in carbohydrate molecules such as sugars and other organic compounds, which are synthesized from carbon dioxide and water. Chlorophyll, the most important class of pigments, assists in this process by trapping solar energy. Confirmatively, we next verified that the improved chlorophyll levels in Mo NPs-exposed tobacco resulted in an increased photosynthetic rate. A previous study demonstrated that CeO_2_ NPs, MoS_2_, ZnO, Fe_2_O_3_, and TiO_2_ NPs stimulated the photosynthetic rates of crops at relatively low exposure levels (Zheng et al., 2005; Alidoust and Isoda, 2013; Tarafdar et al., 2014; Cao et al., 2017; Li et al., 2018; Sun et al., 2020). It was also reported that other engineering nanomaterials, such as CuO, Cu, MoO_3_ and CeO_2_ NPs, could exert a negative impact on photosynthesis at high concentrations by reducing the chlorophyll and carotenoid contents in rice, soybean, corn, and tomato (Du et al., 2015; Yang et al., 2020b; Rai et al., 2021; Sutulienė et al., 2023), or as by damaging photosynthesis-related genes (Wang et al., 2016). The underlying mechanism of this facilitating effect may be attributed to the enhancement of photosynthetic pigments and the function of NPs in regulating biological processes such as stomatal aperture, the synthesis of photosynthesis-related enzymes, enzymatic reactions, and water uptake.

When plants are stimulated by exogenous biotic or abiotic sources, for example, environmental factors, such as plant hormones, metal ions, relative reactive oxygen species (oxygen ions, peroxides, oxygen-containing free radicals, etc.) will be produced in various parts of the plant, resulting in the oxidation of biomolecules such as lipids, proteins, and DNA, and causing cell death (Taran et al., 2016). Hence, ROS-mediated oxidative pressure in plants is usually an effective indicator of phytotoxicity induced by ENMs. Subsequently, plants perceive that their tissues are threatened, which induces the activation of important defense enzymes, such as SOD, POD, and CAT, to eliminate ROS species in plants (Taran et al., 2016). SOD is highly efficient in eliminating superoxide radicals (O_2_^−^) to produce O_2_ and H_2_O_2_ in plants. CAT and POD can directly convert H_2_O_2_ into H_2_O and O_2_ through disproportionation and hydrolysis reactions, respectively (Imada et al., 2016). As has been reported previously, plants exposed to ENMs exhibit significantly increased defense enzyme activities compared to water-sprayed control plants but reduced MDA contents, especially under abiotic stress conditions, such as UV-B radiation, well-watered and drought stress (Aghdam et al., 2016; Imada et al., 2016; Venkatachalam et al., 2017; Sun et al., 2020). Similar changes were observed in other studies showing higher antioxidant enzyme activity of pea at 50 and100 ppm Mo NPs but lower levels of H_2_O_2_ and MDA (Taran et al., 2016; Sutulienė et al., 2021) The results of this study show that after Mo NPs treatment, the activities of three antioxidant enzymes in tobacco seedlings were enhanced in a concentration dependent manner to different degrees, especially after root irrigation treatment. Combining the results in **Figure 3**., it can be observed that Mo NPs not only display strong promotion effects on tobacco plants, but can also serve as an elicitor to stimulate the plant defense system against ambient stress.

Similar results have been reported in previous studies where ZnO NPs significantly improved the leaf total soluble protein contents in green peas and cotton even at lower concentrations (Mukherjee et al., 2016; Venkatachalam et al., 2017). Activating the expression level of stress proteins could possibly explain these results, which is also a response mechanism in plants for protecting their cells from potential oxidative damage caused by NPs (Zhao et al., 2012). Similarly, soluble sugars, especially sucrose and glucose, might also play central roles in higher plant defense responses under biotic and abiotic stresses (Couée et al., 2006). Most importantly, sugar signaling and sugar-modulated gene expression are related to maintaining reactive oxygen species balance in various metabolic reactions, such as mitochondrial respiration or photosynthesis regulation (Koch, 1996; Koch, 2004; Couée et al., 2006). Therefore, it can be speculated that the increase in the soluble sugar levels in tobacco plants after treatment with Mo NPs could be emerging as a signaling molecule that is, conducive to the strength of plant innate immunity.

NPs can usually be absorbed by the leaves or roots of plants and are mainly transported through the xylem and phloem pathways, followed by localization and accumulation in various plant tissues, potentially regulating plant physiological and biochemical processes (Gui et al., 2015). It is implied that both treatment measures accelerated Mo uptake in tissue, however, it seems that the root irrigation treatment shows weaker transport element capacity and causes Mo retention in the root system (**Figure 6**). The same phenomenon was found in another study, where a strong accumulation of Mo in pea roots watered with Mo NPs was determined (Sutulienė et al., 2021). The reason for this may be related to the agglomeration of NPs in soil, which may allow them to pass through narrow vascular tissues in the stem with great difficulty (Collin et al., 2014). Yang et al. found that Mo mainly accumulated in soybean leaves when added to soil (Yang et al., 2020a). Additionally, in most described cases of NPs uptake by plants, either under hydroponic conditions or soil amendments, and with either foliar or irrigation application, it was clear that the absorption and distribution of ENMs by plants were very distinct according to the crop species, soil texture, NPs size, surface charge, and morphology, especially the differences in dispersion (Lv et al., 2019). For example, after ZnO NPs were absorbed by cluster bean and mung bean plants, the released Zn^2+^ was used as a cofactor in the catalytic activity center of phosphatase and improve the activities of phosphatase and inositol hexaphosphatase, consequently the absorption rate of soluble phosphorus in plants increased by 84-108%, and the absorption rate of insoluble phosphorus that is difficult for plants to absorb increased by 10.8% (Raliya and Tarafdar, 2013; Raliya et al., 2016). Therefore, we speculate that Mo NPs can also carry macronutrient elements into the plant during the process of accelerated plant water absorption, thus promoting plant growth and development. This is because Mo is an important component of nitrate reductase and dehydrogenase, which are involved in nitrogen assimilation and transport in higher plants (Kovacs et al., 2015). These enzymes are the key enzymes for plants to convert nitrate nitrogen into ammonium for the synthesis of amino acids and proteins. At the same time, Mo participates in the conversion of inorganic phosphorus to organic phosphorus in plants. When tobacco seedlings absorb NPs through roots or stomata, Mo will inevitably be released into the plant, contributing to the absorption of water and nutrient elements, enhancing photosynthesis, and finally promoting the biomass accumulation of tobacco seedlings (Li et al., 2018).

For most plant species, lignification has been regarded as a basal defense mechanism in the plant immune response that can function as a defensive physical/chemical barrier to external environmental and biotic stress, especially in preventing phytopathogen invasion and colonization (Adhikari et al., 2013; Dhoke et al., 2013; Zhang et al., 2019). Importantly, compared to the control, many more lignifying cells in roots treated with irrigation of Mo NPs than with foliar spraying were observed. Most likely, Mo NPs were identified as exogenous substances, thus activating the defense response in tobacco plants. This reflects the observation that tobacco seedlings seem to respond more sensitively to Mo NPs under root irrigation than spraying, which may be due to roots exhibiting higher NP or metal uptake and accumulation, as indicated in the above results (**Figure 6**). Previous studies have suggested that plant roots are more responsive to ENMs as a result of the higher accumulation of NPs compared with leaves and stems, even under soil-grown conditions (Chen et al., 2022). Our results also showed that the application of Mo, especially irrigation exposure, promoted the differentiation of vascular tissue in stems and leaves and enhanced the leaf vein areas, which will be beneficial to plant growth. Leaf veins have the physiological function of transporting water and nutrients (Hu et al., 2005). Studies have shown that the development of the vascular bundle directly affects the transport of water, inorganic salt *via* xylem and photosynthates *via* phloem, and determines the yield of crops (Yu et al., 2010). These changes increase the flow of water and nutrient elements from roots to leaves after Mo NPs irrigation exposure, and further improve the transportation capacity of vascular tissue, significantly promoting the growth of tobacco seedlings.

Further study was conducted with the assumption that the promotional effects on tobacco in the presence of Mo NPs were ultimately associated with gene regulation at the molecular level. AQPs are integral membrane proteins that increase the permeability of membranes to water as well as to other small molecules such as CO_2_ and glycerol, which play a key role in maintaining plant water balance and water use efficiency. We found that all PS- and AQP-related genes were upregulated in the tobacco plants exposed to Mo NPs by irrigation in comparison to the control plants. Commonly, the activity of AQPs can be impacted by many stress factors such as drought, heavy metals, salinity, pH, and anoxia (Tyerman et al., 1999). Under NPs stresses, some important stress-signaling pathways can be regulated in response to the uptake of NPs, which could induce physiological processes in plants (Sun et al., 2020). The tested AQP genes encode plasma membrane intrinsic proteins (PIPs). *NtAQP1*, a stress-induced gene, plays vital roles in controlling the CO_2_ and water permeability of mesophyll tissues in tobacco plants, in addition to increasing the photosynthetic rate, stomatal opening, and leaf growth, especially under abiotic stress. *NtPIP2;1* has also been reported to have high water transport activity. The increased transcription levels of *NtPIP2;1* in tobacco tissues showed that the irrigation application of Mo NPs significantly promoted water uptake, and indicated the moderate increase in the water content in tobacco after foliar spraying with 100 μg/mL Mo NPs. A similar study found that the water-channel protein (LeAqp2) was upregulated in tomato root tissues exposed to MWCNTs (Khodakovskaya et al., 2011).

Extensive studies have been performed regarding the effects of different nanomaterials on crop growth under different application methods. Some findings are contrary to the results of this study. Adhikari et al. found that the length of roots was obviously inhibited after rice exposure to Mo NPs at concentrations greater than 5 mg/L (Adhikari et al., 2013). Interestingly, our findings demonstrated that exposure to 100 mg/L Mo NPs still promoted tobacco plant growth without any phytotoxicity in either case. For metal oxide NPs, compared to soil amendment, Fe_2_O_3_ NPs more significantly enhanced the biomass and photosynthetic rates of soybean when foliar sprayed, which was attributed to the increase in stomatal opening (Alidoust and Isoda, 2013). Some studies on plant exposure to ZnO NPs indicated that ZnO NPs suppressed wheat root growth and reduced the biomass under root irrigation in field soil and sandy soil, which mostly depended on the continuous uptake of many zinc ions released from NPs, thus inducing the production of oxidative stress and causing biological toxicity (Du et al., 2011; Dimkpa et al., 2012). However, the foliar spraying of ZnO NPs facilitated the growth and productivity of wheat, tomatoes, and mung beans (Dhoke et al., 2013; Raliya et al., 2015). These different results may be due to differences in experimental methods, NPs properties, plant species, and the nutrient absorption and metabolic ability of plants at different growth stages.

According to our previous investigation, the soil available Mo content in the Chongqing tobacco growing area was generally low (0.05-5 mg/kg), mainly due to nutrient loss caused by long-term soil acidification (He et al., 2005; Kaiser et al., 2005). Unlike other metal ions, Mo usually exists in soil as organic compounds or water-soluble molybdate ions (MoO^4-^) and ammonium molybdate ions (NH_4_Mo^2+^) and participates in the life activities of plants (Gupta and Lipsett, 1981). At present, the use of nanotechnology to improve the disease resistance and vegetative growth of flue-cured tobacco has achieved initial results that are of great significance for reducing fertilizer input and ecological and environmental protection (Chen et al., 2022). This study was carried out in potting substrates and demonstrated that Mo NPs can promote the growth of tobacco seedlings and the synthesis of chloroplasts and proteins, which is largely dependent on the uptake of Mo (Raven, 1988). Therefore, Mo NPs not only have good biocompatibility but can also be expected to be used as micronutrient fertilizers for plant nutrition. However, the biological effects of Mo NPs in soil and the migration behavior of Mo NPs in plants are still unclear and need to be further explored.

## 5 Conclusion

The aim of this study was to clarify the effects of Mo NPs on tobacco growth under two exposure modes. The results demonstrated that the biological effects of Mo NPs applied by irrigation were superior to those obtained in response to foliar exposure, which exerted significantly positive effects on tobacco seedling growth, resulting in a remarked physiological and biochemical changes. After soil application, more Mo accumulated in stems and roots compared to that observed under foliar spraying, especially 100 mg/L Mo NPs exposure. The increase in the defensive enzyme activity and upregulation of eight genes involved in the photosynthesis process and aquaporins indicated enhanced disease resistance. This pattern on the increased Mo content suggests that Mo NPs may be absorbed by tobacco plant, although more experiments are needed to confirm it. Therefore, the transport kinetics of nanoparticles and location in plant tissues still need to be further clarified. And what’s more, the necessity of the Mo NPs environmental risk assessment must be emphasized before wide use in agriculture. Firstly, it is necessary to evaluate the biological effects of Mo NPs based on a wider range of plant species. Secondly, more studies on the effects of Mo NPs on soil microbial communities are also required to consider the use of Mo NPs as potential nano nutritional supplements, especially whether they interfere with soil beneficial microbial composition and function.

## Data availability statement

The original contributions presented in the study are included in the article/**Supplementary Material**. Further inquiries can be directed to the corresponding author.

## Author contributions

JC, conceptualization, writing the original draft. YY, KS, and YZ, performed the experiments and statistically analyzed the data. WD, funding acquisition, English editing, revised preparation. All authors contributed to the article and approved the submitted version.
