# Corrigendum: Favorable physiological and morphological effects of molybdenum nanoparticles on tobacco (*Nicotiana tabacum* L.): root irrigation is superior to foliar spraying

**DOI:** 10.3389/fpls.2023.1320236

**Published:** 2023-11-03

**Authors:** Juanni Chen, Ying Yin, Yunsong Zhu, Kun Song, Wei Ding

**Affiliations:** Laboratory of Natural Product Pesticides, College of Plant Protection, Southwest University, Chongqing, China

**Keywords:** Mo NPs, tobacco, root irrigation, stimulating growth, morphological effects

In the published article, there was a mistake in the Acknowledgments. The statement for the Key Science and Technology Project of Sichuan Tobacco Company (SCYC202114), Science and Technology Project of Sichuan Provincial Company of China National Tobacco Corporation (SCYC202114) was displayed. The correct statement is “Key Science and Technology Project of Chongqing Tobacco Company (B20211NY1316), Science and Technology Project of China National Tobacco Corporation (110202101047 (LS-07))”. In the published article, “The authors declare that this study received funding from Chongqing Tobacco Company. The funder was not involved in the study design, collection, analysis, interpretation of data, the writing of this article or the decision to submit it for publication” was not included. The correct statement appears below.

**“**The authors acknowledge the financial support by National Natural Science Foundation of China (32001934); Key Science and Technology Project of Chongqing Tobacco Company (B20211NY1316), Science and Technology Project of China National Tobacco Corporation (110202101047 (LS-07)); Science and Technology Project of Liangshan Prefecture Company of Sichuan Provincial Tobacco Company (202051340024416).

The authors declare that this study received funding from Chongqing Tobacco Company. The funder was not involved in the study design, collection, analysis, interpretation of data, the writing of this article or the decision to submit it for publication”.

The authors apologize for this error and state that this does not change the scientific conclusions of the article in any way. The original article has been updated.

