# Supplementary material for: Favorable physiological and morphological effects of molybdenum nanoparticles on tobacco (Nicotiana tabacum L.): root irrigation is superior to foliar spraying
Source: Front Plant Sci. 2023 Aug 31;14:1220109. doi: 10.3389/fpls.2023.1220109 (PMC10501311; doi:10.3389/fpls.2023.1220109)
Supplement: Supplementary file 1 [file DataSheet_1.docx]

**Favorable physiological and morphological effects of molybdenum nanoparticles on tobacco (*Nicotiana tabacum* L.): root irrigation is superior to foliar spraying**

**Juanni Chen, Ying Yin, Yunsong Zhu, Kun Song, Wei Ding***

Laboratory of Natural Product Pesticides, College of Plant Protection, Southwest University, Chongqing 400715, China；

*** Correspondence:**

Wei Ding

dingw@swu.edu.cn

Tel.: +86-23-6825-0953; Fax: +86-23-6825-0953

**2.2 Seed germination assay**

The effects of MoNPs on seed germination was determined as described in the previous research. Firstly, tobacco seeds were surface sterilized with 10% NaClO solution for 15min, followed by a rinse thorough with sterilized deionized water for five times at least. After that, the 30 of tobacco seeds were evenly immersed in MoNPs suspension filled in standard Murashige and Skoog (MS) medium containing GO at 22℃ in fully sterile Petri dishes (90 mm in diameter) at different concentrations, respectively. All the dishes were transferred to artificial climate chamber (PRX-450C, Saifu, China) with the temperature of 28℃ (80-90% relative humidity) and incubated for 12h in complete dark. The criterion for seeds germination was indicated as the emergence of a radicle (1−2 mm). The experiment was repeated three consecutive times. After incubation for 5 d, the germination rate were calculated.


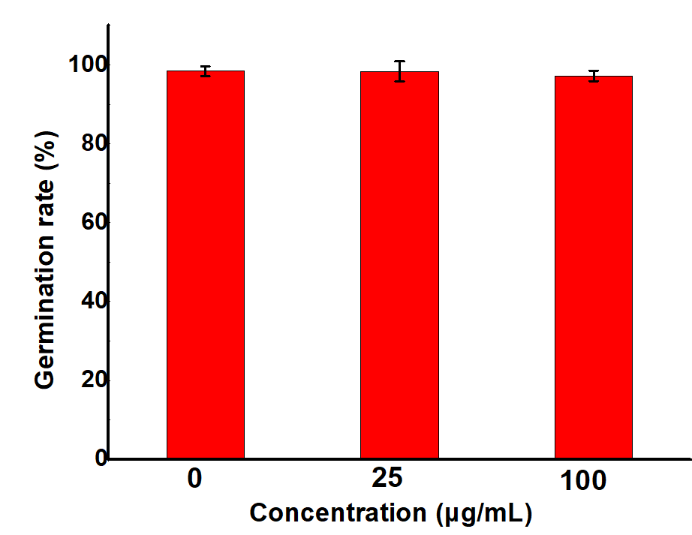


**Figure S1** Germination rate of tobacco seeds exposed to different doses of MoNPs (0, 25 and 100 μg/mL).


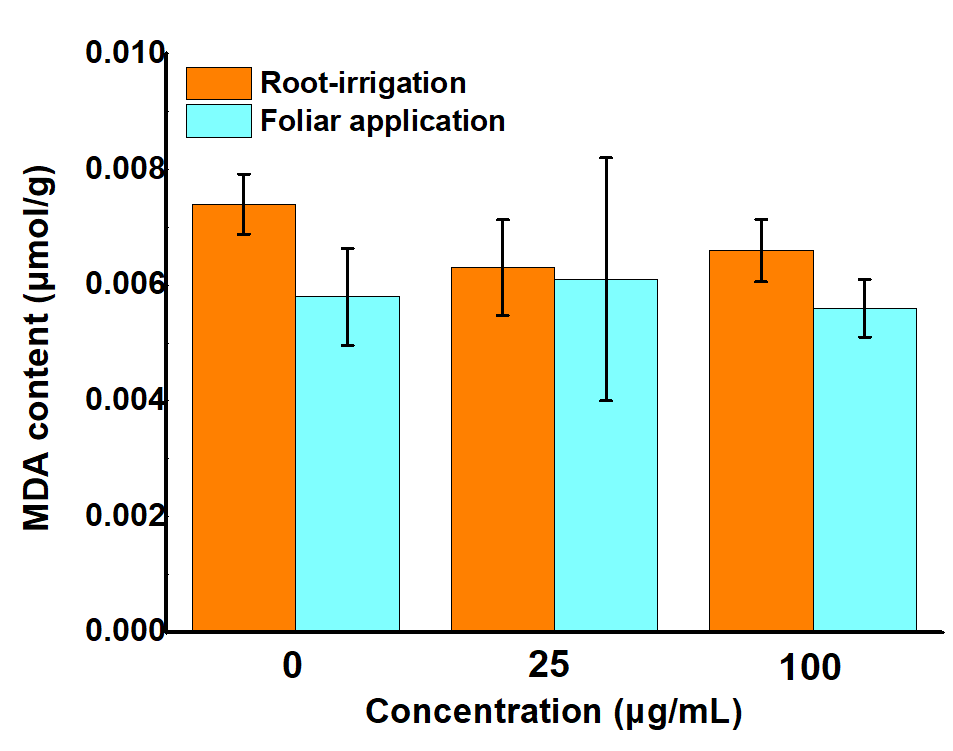


**Figure S2** MDA content of tobacco leaves exposed to different doses of MoNPs (0, 25 and 100 μg/mL) applied by root-irrigation and foliar spraying for 25 days.


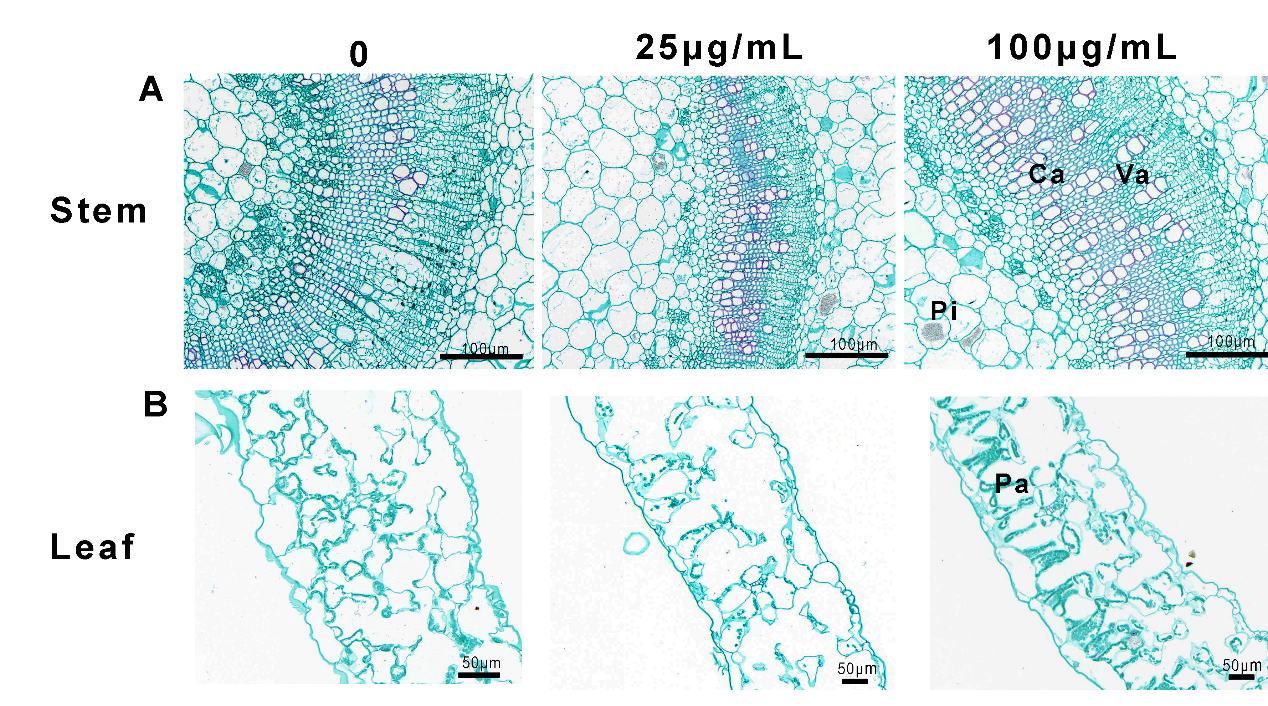


**Figure S3** Morphological structures observed by an optical microscope of tobacco stem (A) and leaf (B) after spraying exposure to MoNPs. The pictures on the right are partial magnification of the image marked with the corresponding numbers. Cross-sections of tissues from the same position of seedlings treated for 25 day were used for this assay. Ca=catheter; Co=cortex; Ep=epidermis; Fi=fibers; Pa=Palisade tissue; Ph=phloem; Pi=pith; St=sieve tube; Va=Vascular bundle; Ve=vein; Xy=xylem;

**Table S1 The composition of fertilizer solution**

| **Chemicals** | **Concentration (mg/L)** |
| --- | --- |
| KNO_3_ | 1900 |
| KH_2_PO_4_ | 170 |
| CaCl_2_·2H_2_O | 440 |
| H3BO3 | 6.2 |
| ZnSO_4_·7H_2_O | 8.6 |
| CuSO_4_·7H_2_O | 0.025 |
| FeSO_4_·7H_2_O | 27.8 |
| Glycine | 2 |
| VB_1_ | 0.5 |
| Na_2_EDTA | 37.3 |
| NH_4_NO_3_ | 1650 |
| MgSO_4_·7H_2_O | 370 |
| KI_2_ | 0.83 |
| MnSO_4_·H_2_O | 22.30 |
| Na_2_MoO_4_·2H_2_O | 0.25 |
| CoCl_2_·6H_2_O | 0.025 |
| Inositol | 100 |
| VB_6_ | 0.1 |
| Niacin | 0.5 |

**Table S2 Primer sequences**

| **Genes** | **Primer sequences** |
| --- | --- |
| *NtActin1* | F: 5'-CCTAGTAAGCGCGAGTCATCAGC-3'  R: 5'-GCGACGGGCGGT GTGT-3' |
| *NtPIP1;1* | F: 5'-CCTTCTAGTGTGTGTCTGTGTGTG-3'  R: 5'-GGCAGGAGAATCTTAGCT CTTG-3' |
| *NtPIP2;1* | F: 5'-AGAAACAATGTCAAAGGACGTG-3'  R: 5'-CTGGTAGTGGTTGCAAAAGTTG-3' |
| *NtTIP1* | F: 5'-ATGCCGATCCACCAAATTG-3'  R: 5'-TTAAAAATCTCCACTGGGGAG TG-3' |
| *NtPIP1* | F: 5'-GGTTCATTTGGCCACCATCCCAAT-3'  R: 5'-GCAGCAAGAGCAGCTCCAATGAAT-3' |
| *psbA* | F: 5'-TCC AGT AGA(C/T)AT TGA TGG TAT TGG-3'  R: 5'-GA(A/G) GTT ACC AA(G/A) GAA CC(A/G) -3' |
| *Lhcb1* | F: 5'-CCGTGARCTWSARGTKATCCA-3'  R: 5'-GARCRAAGAAYCCRAACATDGA-3' |
| *rbcL* | F: 5'-GTTTGGACCGATGGACTTAC-3'  R: 5'-TCGAAGCTGCCACCTGGATA-3' |
| *rbcS* | F: 5'-ATGGCTTACTCTATGCTCTCCTC-3'  R: 5'-TTAAGCACCGGTGAAGCTTGG-3' |
